# Human resource capacity for information management in selected public healthcare facilities in Meru County, Kenya

**DOI:** 10.11604/pamj.2015.20.334.6052

**Published:** 2015-04-07

**Authors:** Elizabeth Mueke Kiilu, Dominic Charles Okero, Lillian Muiruri, Pacific Akinyi Owuondo

**Affiliations:** 1 Meru Level Five Hospital, Githongo District Hospital, GK Prison health center & Kinoru Dispensary in Meru County, Kenya; 2 Department of Health Systems Management, Kenya Methodist University, Nairobi, Kenya

**Keywords:** Health worker capacity, information management

## Abstract

**Introduction:**

Reliable health information is essential for decision making in the healthcare system. Information management in Kenya was considered the weakest area under the Health Information System pillar mainly due to inadequate health workers capacity. The study therefore aimed at assessing health workers skills and current training needs for information management in the selected healthcare facilities.

**Methods:**

Cross-section research design was adopted and both purposive sampling technique and censuses were used to establish the study participants. Analysis was done using SPSS version 20 and results were presented in tables, charts and graphs.

**Results:**

It was established that capacity building was usually undertaken through on-job trainings i.e. 85.1% (103) health workers had on-job training on filling of data collection tools and only 10% (13) had received formal classroom training on the same. Further, only 9.1% (11) health workers had received information management training while 90.9% (110) had not received such training. Health workers demonstrated below average skills on information management i.e. only 17.4% (21) could check for data accuracy, only 16.5% (20) could compute trends from bar charts and only 16.5% (20) could transform the data they collected into meaningful information for use.

**Conclusion:**

The researcher recommended that healthcare facilities management teams develop a competency based framework for defining the desired skill mix for information management and have a yearly Training Needs Assessment for assessing training needs for information management among the health workers.

## Introduction

For effective coordination of healthcare activities, high quality information is crucial and this is generated and managed under the pillar of Health Information System (HIS). Properly organized HIS is a prerequisite for the efficient administration of health services and their planning should be given high priority [1]. Globally, there is a rigorous demand for HIS so as to ensure a well functioning health system, especially because of increased accountability for resource allocation and the need for measuring health outcomes. Further, for donors and decision-makers to invest financially in HIS, it is important that performance requirement of the generated information is of high quality, accurate, complete and reliable [1]. World Health Organization (WHO) [2] revealed that there were comparatively few papers discussing the shortage of HIS skills among the general health personnel and even fewer papers which actually described the training needs requirement and the ideal distribution of the skills across the various healthcare facilities on information management. Kenya has had a long history of health sector reform with HIS being established in 1984 in each district thereby, decentralizing health data and information processing to the facility level. However, more investment was required to adequately sustain HIS operations especially in the peripheral health units where data collection registers were often improvised and reporting forms not always available. This caused inaccurate data entry which was untimely and often incomplete compromising the quality of information collected and its consequent underuse by the various stakeholders [3]. Currently the HIS Department at the ministry of health in Kenya is grossly understaffed with only 11% of the human resource requirements in post. The major gaps were in the cadre of Health Records and Information Officers (gap of 88.3%), Information, Communication and Technology (ICT) officers (gap of 96.6%), and statisticians (gap of 100%) [4]. The Kenya HIS policy therefore stated that in the absence of specialized HIS personnel, the general health workers must have the basic information management skills which included the ability to accurately undertake data collection, information analysis and interpretation. Further, the Government of Kenya report [3] pointed out that data and information management in Kenya was considered as the weakest area under the HIS pillar. This was attributed by inadequate skills on information management and training of health workers that was not focused towards information management activities. Additionally, poor information management was worsened by inadequate supply of data collection tools, computers, software and hardware for Information, Communication and technology, insufficient storage space and security for information collected within the healthcare facilities. Further human resource mapping done in Kenya in 2004, showed that 46% of staff working on HIS tasks had no professional training on Health Information System Management, thus compromising the capacity of the healthcare institutions to generate quality information for policy, planning and monitoring of the healthcare system activities [3]. In Meru County, there was lack of scientifically sound information on the general health workers skills for information management which led to barriers for effective human resource planning for HIS workers in the County. Likewise, there was lack of information on health workers training needs for information management in the healthcare facilities. Without adequate knowledge on training needs, it was found to be impossible to practically develop a customized program for resolving knowledge gaps on information among the health workers.

## Methods

**Study sites**: The study was conducted at Meru Level Five Hospital, Githongo District Hospital, Kinoru Dispensary and GK Prison health center in Meru County, Kenya. These facilities were chosen because they were high volume facilities and main referral facilities in their respective locations, hence were seen as a representation of the facilities in Meru County.

**Study design**: Cross-sectional design was used. It was used because it provides a snapshot of the exposed variables across a wide population without manipulating or influencing the study population in any way. Cross-section design was also used because it could produce multiple outcomes and various variables could be studied at a given point in time.

**Data collection**: Both open-ended and closed-ended, structured questionnaires which were self administered were used to collect data. An observation check-list was also used to assess the work environment for conducting information management activities in the selected facilities in Meru County.

**Data management and analysis**: The data obtained from this study was subjected to statistical analysis using descriptive statistics. After collection of data, it was edited, coded and examined for errors and omissions then corrected appropriately. In the coding process, data was organized into categories after which, numerals were assigned to each item prior to entering them into the computer for storage. After entering using the Statistical Package for Social Statistics (SPSS) program version 20, the data was subjected to descriptive and qualitative analysis. Correlation coefficient was used to establish relationships between the variables. The results were then presented using tables, graphs and pie charts, besides narrative descriptions.

**Ethical considerations**: Permission was obtained from the Medical Superintended and the hospital research committee in Meru Level Five Hospital and Githongo District Hospital. The researcher maintained a high level of confidentiality as the data collected contained personal information about the population researched. Additionally, the purpose and the expected outcome of the study were explained in a simple and honest way to the participants in the study.

## Results

Four public healthcare facilities were engaged during this process over a period of four months (from July 2014 to October 2014) in Meru County, Kenya.

**Figure 1:**
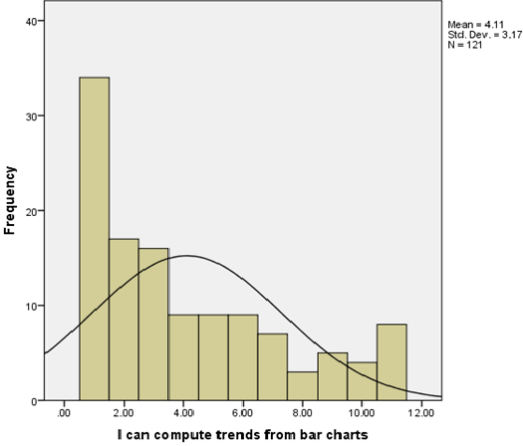
Ability to compute trends from bar charts in Meru County, Kenya 2014

**Figure 2:**
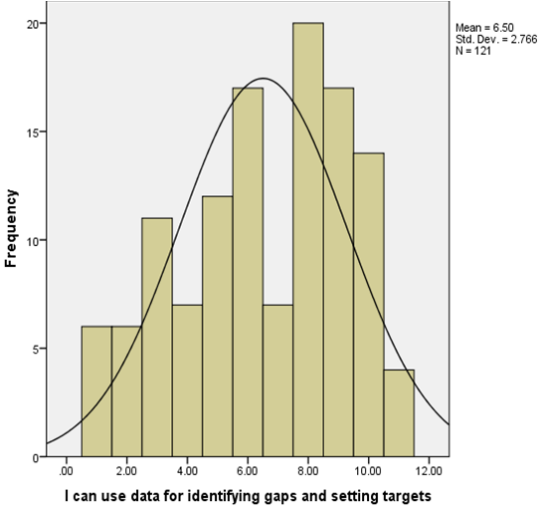
Use of information for identifying gaps and setting targets in Meru County, Kenya 2014

**Figure 3:**
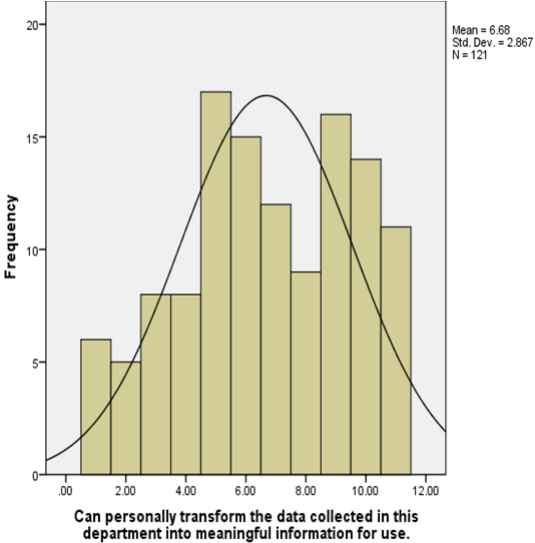
Ability to change data into information in Meru County, Kenya 2014

**Health workers skills for information management**: Health workers knowledge of computer use was found to be insufficient i.e. that majority of the respondents 62 (51.2%) had knowledge on computer use while 58 (47.9%) of the respondents had no knowledge on how to use computers. Health workers were also further tested on their ability to use a computer for information management at their various work stations and the findings were a follows; majority 51 (42.1%) of the respondents rated themselves as intermediate users, 11 (9.1%) were new or inexperienced users and only 6 (5.0%) were experienced users. Further, majority of the respondents 67 (55.4%) of the respondents did not know about HIS while 52 (43%) knew about it. The respondents ability to check for data accuracy was tested and the data collected indicated that majority 28 (23.1%) of the respondents had little idea on checking for data accuracy, 26 (21.5%) were competent, 25 (20.7%) had no idea on how to check for data accuracy, 21 (17.4%) were average, and 21 (17.4%) were above average. Ability to compute trends from bar charts was also assessed i.e. (Figure 1). Respondents’ ability to use information for identifying gaps and setting targets was also assessed as shown in (Figure 2). The ability to transform data into meaningful information for use at the department level showed that majority of the respondents were unable to perform this function (Figure 3).

**Figure 4:**
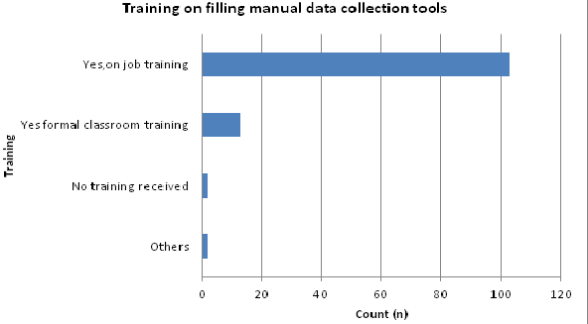
Training on filling of manual data collection tools in Meru County, Kenya 2014

**Health workers training needs for information management**: Source of computer knowledge was assessed to determine if health workers had received formal training on the same. It was found that majority 34 (28.1%) of the respondents gained their computer knowledge from formal classroom, 22 (18.2%) from informal set-up, 11 (9.1%) of the respondents had gained their knowledge from on-job training while 1 (0.8%) of respondents gained from other unspecified sources. Training on filling of manual data collection tools was also majorly done through on-job training rather than through formal classroom training (Figure 4). Training on information management was found to be inadequate with majority 110 (90%) of the respondents having not received any such training, while only 11 (9.1%) having been trained on information management. Further, respondents were asked to indicate their willingness to attend a four week training on information management. The data collected indicated that majority 97 (80.2%) of the respondents would be willing to attend the training while 16 (13.2%) would not be willing to attend the four week training. Factors influencing training attendance among the health workers was also assessed i.e. (Table 1). Correlation co-efficient between the dependent and independent variables was done and results were as seen in (Table 2).

**Table 1:** Factors influencing training attendance among health workers in Meru County

	Tuition costs	Location of Training	Duration of Training	Per-diem offered (daily allowances)
Not Important	5 (4.1%)	17 (14%)	8 (6.6%)	4 (3.3%)
Little Important	2 (1.7%)	16 (13.2%)	10 (8.3%)	6 (5%)
Moderately	8 (6.6%)	18 (14.9%)	20 (16.5%)	6 (5%)
Important	25 (20.7%)	40 (33.1%)	44 (36.4%)	32 (26.7%)
Very Important	81 (66.9%)	30 (24.8%)	39 (32.2%)	72 (60%)

**Table 2:** Correlation of the dependent and independent variables

Independent Variables		Health workers skills	Health workers training needs
**Information Management in Meru County (Y)**	Pearson Correlation (r)	305*	528*
	Sig. (2-tailed)	001	004

## Discussion

Health worker skills and knowledge on information management was assessed to determine their ability to handle health information. Government of Kenya [5] report noted that there was little attention paid to the quality of information produced in the country and that the general health workers were not adequately skilled to handle health information. Miriam, Vicki and Maxine [6] say that the general skills required for health workers to manage data and information effectively include the ability to analyze the data that they collect, so that they can use it to make meaningful decisions from the information generated. Data analysis required basic knowledge on computer use. However from the data collected only one-half of the health workers had knowledge on computer use. However in many of the Lower and Middle Income Countries (LMIC) settings HIS is often paper based e.g. in a study conducted in Malawi deduced that only 25% of the health facilities had a computer and none of the centers had internet [7]. This greatly affected the quality of information produced from the healthcare system. Further, majority of the health workers 55.4% had no knowledge on HIS. These findings concurred with Human resource mapping done in Kenya in 2004 that showed that 46% of the staff working on health information tasks had no professional training on the same, thus compromising the capacity of the healthcare institutions to generate quality information for policy, planning and monitoring of the healthcare system activities [5]. Health workers were also assessed on their ability to perform basic information management tasks such as their ability to check for data accuracy, ability to use information for identifying gaps and setting targets, and their ability to compute trends from bar charts. The data collected indicated that majority of the health workers were unable to perform these basic information management functions. These findings were consistent with the findings of Government of Kenya [3] review undertaken on HIS which indicated that while the basic data capture and reporting skills were present, there was little attention to paid to information quality and health care workers were not competently trained to undertake most of the information management activities i.e. checking for information quality (which includes checking for data accuracy, timeliness, completeness and consistency), data analysis and the interpretation of health information. Further, Maxine, Renata, & Anna [8] put forward that the general skills required for health workers included the ability to understand and use primary and secondary information, be able to document accurately medical records to ensure quality information and finally, understand policy and procedures for data collection, management and analysis. Computing of trends from bar charts would enable health workers to check for disease trends over a given period of time and compare disease outcomes with the interventions for disease management put in place. This skill would also facilitate planning for disease management and possible disease outbreaks among other functions. Government of Kenya [4] demonstrated that health workers had inadequate requisite skills at all levels for information management therefore creating an exceptionally low level of commitment from health workers for information management.

Health workers were also assessed on trainings undertaken on information management so as to identify any training gaps on the same. The data collected indicated that majority of the respondents had not received formal trainings and that knowledge on information management functions was usually gained through informal set ups or on job trainings. In a study conducted in Lesotho, the situation was also found to be similar and recommendations given for the study by Miriam, Vicki, & Maxine [8] was that continued training/ education should be two pronged, where existing health workforce would be skilled through short-term training programs while others would benefit from long-term training for re-professionalization. Further majority of the respondents demonstrated a willingness to attend training on information management which clearly pointed out that majority of the health workers felt that there was knowledge gap on information management and that they were willing to attend a training to fill the knowledge gap. Since majority of the health workers were willing to attend trainings on information management, some of the factors influencing training attendance were also assessed. This would shed light for policy makers on factors to consider when planning for trainings for health workers so as to improve attendance and ensure maximum knowledge is gained from these trainings. Some of the factors influencing training attendance that were assessed included tuition costs, location of training, duration of training and per-diems offered (daily allowances) as shown in (Table 1). For instance, when respondents were asked how they felt about tuition costs majority 116 (87.6%) of the respondents would attend trainings only if the tuition costs were catered for and only 5 (4.1)% would not mind to cater for their own tuition costs. This was important for policy makers to note for purposes of planning for information management training activities for health workers and ensure that training costs were catered for to improve attendance and motivation to attend training on information management.

The respondents were also asked to indicate how they viewed location of training, the data collected showed that majority of the respondents 70 (57.9%) preferred to have the trainings conducted near their work places and not have to travel to distant areas for training. This fact was also supported by other authors AbouZahr & Boerma [9] who stated that trainings should be conducted within the country whenever possible, to minimize the need for essential staff to leave their posts, and to reduce the burden and cost of training oversees. When asked about the duration of training, majority of the respondents 83 (68.6%) preferred short term trainings as compared to full time long courses. However AbouZahr & Boerma [9] recommend that training should be appropriate for different capability levels with specific training paths identified ranging from short-term workshop and full oversees degree options. Finally the respondents were asked to indicate their take on per-diem offered (daily allowances) during the period of training and the data collected indicated that majority of the respondents 104 (85.9%) viewed per-diem offered as an important factor when being trained on information management skills and viewed it as an incentive for attending trainings for information management. Quantitative analysis was also conducted to determine the correlation of the dependent and the independent variables as depicted in (Table 2). The findings were as follows; there was a weak positive relationship between health workers skills and information management in Meru County as indicated by correlation of 0. 305. The p-Value of 0.001 was less than the acceptable significance level (α), hence the null hypothesis that there was no relationship between health workers skills and information management in Meru County was rejected. This showed that the sampled data could be applied to the general population at 95% confidence level. There was also a weak a weak positive relationship between health workers training needs and information management in Meru County as indicated by correlation of 0. 528. The p-Value of 0.004 was less than the acceptable significance level (α), hence the null hypothesis that there was no relationship between health workers training needs and information management in Meru County was rejected. This showed that the sampled data could be applied to the general population at 95% confidence level.

**Limitations of the assessment**: During the research period, not all the targeted health workers might be available to respond to the questionnaires. The researcher therefore extended the data collection period so that more respondents could be captured over a longer duration. The study was conducted in high volume facilities and the main referral facilities within their regions and was therefore seen as a representation of health care facilities in Meru County.

## Conclusion

The study established that majority of the health workers had between below average and average skills on information management except for the staff in the health records department who demonstrated above average skills. Additionally, health workers had deficits in training on information management especially in the areas of checking for data accuracy, computing of trends from bar charts, data analysis and use of the information generated to make decisions at the department level.

**Recommendations**: As a contribution to the strengthening of the HIS pillar in Meru County, several evidence based recommendations were suggested. The researcher recommended that the Meru County and sub-County Health Management Teams should; develop a Competency Based Framework for defining the required skills for proper information management among health workers at the various levels. Additionally, they should also develop a yearly Training Needs Assessment (TNA) to assess the training needs requirements for information management hence identifying the training needs gaps and tailor training based on these gaps.
